# MyI-Net: Fully Automatic Detection and Quantification of Myocardial Infarction from Cardiovascular MRI Images

**DOI:** 10.3390/e25030431

**Published:** 2023-02-28

**Authors:** Shuihua Wang, Ahmed M. S. E. K. Abdelaty, Kelly Parke, Jayanth Ranjit Arnold, Gerry P. McCann, Ivan Y. Tyukin

**Affiliations:** 1 Department of Cardiovascular Sciences, University of LeicesterGlenfield Hospital, Leicester LE3 9QP, UK; 2 The NIHR Leicester Biomedical Research Centre, Glenfield Hospital, Leicester LE3 9QP, UK; 3 School of Computing and Mathematical Sciences, University of Leicester, Leicester LE1 7RH, UK; 4 Department of Mathematics, King’s College London, London WC2R 2LS, UK; 5 Department of Geoscience and Petroleum, Norwegian University of Science and Technology, 7491 Trondheim, Norway; 6 Department of Automation and Control Processes, Saint-Petersburg State Electrotechnical University, 197022 Saint-Petersburg, Russia; 7 Laboratory of Advanced Methods for High-Dimensional Data Analysis, Lobachevsky University, 603105 Nizhni Novgorod, Russia

**Keywords:** MyI-Net, myocardial infarction, automatic detection, deep learning, MRI

## Abstract

Myocardial infarction (MI) occurs when an artery supplying blood to the heart is abruptly occluded. The “gold standard” method for imaging MI is cardiovascular magnetic resonance imaging (MRI) with intravenously administered gadolinium-based contrast (with damaged areas apparent as late gadolinium enhancement [LGE]). However, no “gold standard” fully automated method for the quantification of MI exists. In this work, we propose an end-to-end fully automatic system (MyI-Net) for the detection and quantification of MI in MRI images. It has the potential to reduce uncertainty due to technical variability across labs and the inherent problems of data and labels. Our system consists of four processing stages designed to maintain the flow of information across scales. First, features from raw MRI images are generated using feature extractors built on ResNet and MoblieNet architectures. This is followed by atrous spatial pyramid pooling (ASPP) to produce spatial information at different scales to preserve more image context. High-level features from ASPP and initial low-level features are concatenated at the third stage and then passed to the fourth stage where spatial information is recovered via up-sampling to produce final image segmentation output into: (i) background, (ii) heart muscle, (iii) blood and (iv) LGE areas. Our experiments show that the model named MI-ResNet50-AC provides the best global accuracy (97.38%), mean accuracy (86.01%), weighted intersection over union (IoU) of 96.47%, and bfscore of 64.46% for the global segmentation. However, in detecting only LGE tissue, a smaller model, MI-ResNet18-AC, exhibited higher accuracy (74.41%) than MI-ResNet50-AC (64.29%). New models were compared with state-of-the-art models and manual quantification. Our models demonstrated favorable performance in global segmentation and LGE detection relative to the state-of-the-art, including a four-fold better performance in matching LGE pixels to contours produced by clinicians.

## 1. Introduction

Myocardial infarction (MI) occurs when an artery supplying blood to the heart is abruptly occluded. This is caused by the rupture of an atherosclerotic plaque in the wall of the artery, triggering a clotting cascade and leading to vessel occlusion. This may result in severe damage to the heart muscle, which may be irreversible (scar). The extent of scarring following more severe MIs (ST segment elevation MI, or STEMI) may drive enlargement of the heart and is associated with worse prognosis (increased risk of death and subsequent heart failure) [1,2]. According to a report from the British Heart Foundation (BHF) in 2020, MI accounts for approximately 100,000 hospital admissions annually. It is estimated that there are 1.4 million individuals alive in the UK today who have survived an MI (1 million men and 380,000 women) [3].

Cardiovascular magnetic resonance imaging (MRI) provides accurate non-invasive diagnosis of MI. The late gadolinium enhancement (or LGE) technique [4,5] uses gadolinium-based contrast agent and specified magnetic resonance pulse sequences to provide a reproducible method for identifying and quantifying MI. LGE-CMR is recognized as the “gold standard” non-invasive method for visualizing and diagnosing MI and also provides vital prognostic information following MI. Several methods are available for the quantitative assessment of MI size (from LGE images), including visual assessment, manual planimetry, and semi-quantitative methods (such as full width at half maximum [FWHM]) [6,7]. However, to date, there is no “gold standard” fully automatic method for MI detection and quantification. 

In the past decades, several groups of researchers have been working to develop either semiautomatic or fully automatic methods for the detection and quantification of MI from MRI scans. For example, Eitel et al. [8] proposed a standard deviation (SD) method for the quantification of the salvaged myocardium area extent after reperfusion. Amado et al. [9] used the FWHM criterion to confirm that MI can be sized accurately up to 30 min after contrast administration. Flett et al. [10] compared seven quantification methods including manual quantification, 2, 3, 4, 5, or 6 SDs above remote myocardium, and the full FWHM method. They confirmed that FWHM methods provide the closest result to manual quantification and have the highest reproducibility. Hsu et al. [11] meaured the MI size of 11 dogs based on automated feature analysis combined with thresholding (FACT). The comparison of the proposed FACT algorithm with FWHM, intensity thresholding and human manual contouring confirmed that human contouring may overestimate MI size and higher accuracy can be obtained from FACT than intensity thresholding. Tong et al. [12] proposed the current interleaved attention network (RIANet) for cardiac MRI segmentation based on ACDC 2017. Shan et al. [13] proposed a segmentation method based on spatiotemporal generative adversarial learning witout contrast agents. Xu et al. [14] proposed a long short-term memory recurrent neural network (LSTM-RNN) for MI detection without contrast agents. Héloïse Bleton [15] proposed left ventricular infarct location based on neighborhood approximation forests (NAF) and compared this with the stack autoencoder method based on 4D cardiac sequences. Fahmy et al. [16] developed a UNet DCNN model for automatic cardiac MI quantification with stratified random sampling. Bernard et al. [17]’s review reported that for the ACDC2017 challenge of cardiac MRI assessment, many researchers proposed using UNet [18] for the segmentation of the myocardium, right ventricle and left ventricle. Fahmy et al. [19] also proposed using the UNet method for MI segmentation based on data collected from patients with and without MI.

Although significant progress has already been made in assisting clinical experts to quantify the size of MI in affected patients, major hurdles still remain in this vitally important area. For example, manual tracing of contours is subjective and prone to low reproducibility with high intra- and interobserver variability, as well as being labor-intensive, with associated costs. Existing semi-automatic methods to localize MI are affected by biases introduced through tracing of the left ventricle (LV). All these challenges introduce significant uncertainty when detecting and quantifying LGE from cardiovascular MRI images. Therefore, in this paper, we propose a new system, named MyI-Net, to achieve end-to-end, fully automatic MI detection and quantification to overcome the above challenges.

The rest of this paper is organized as follows: Section 2 provides a detailed account of materials, procedures of the data collection and the demographics of data. Section 3 presents our proposed methods, including the construction of the weight matrix, details of data augmentation, and performance metrics. Section 4 presents the results of our experiments, including time cost analysis, segmentation analysis and comparison with the state of the artwork as well as manual segmentation produced by human experts. Section 5 concludes and illustrates our proposed method, its limitations and future research.

## 2. Materials

The data were collected using cardiovascular magnetic resonance (CMR) imaging. With gadolinium-based contrast agents and appropriate pulse sequences, CMR can provide clear differentiation between infarcted and normal myocardium. To obtain LGE images, the patient is typically scanned 10–20 min after the intravenous administration of a standardized, weight-adjusted dose of gadolinium-based contrast agent. 

The data came from a variety of MRI scanners: for data collected from Siemens 1.5T scanners, the sequence parameters were as follows: slice thickness was 10 mm, repetition time was 900 ms, echo time was 4.91 ms, flip angle 30o and Acquisition matrix—256/154. For Philips 1.5T, slice thickness was 10 mm, repetition time was 4.87 ms, echo time was 1.87 ms, acquisition matrix—256/256. For Siemens 3T Skyra, slice thickness was 8 mm with a 2 mm gap, repetition time was 43.29 ms, echo time 1.46 ms and acquisition matrix—256/208. These are standard parameters used for routine clinical examinations, which are in accordance with the published [20]. The added advantage of exploiting data from different vendors and field strengths is that it has the potential to facilitate the generalization and robustness of the model. The data were collected from patients with MI whose demographic data are shown in Table 1.

## 3. Automated Segmentation of Myocardial Infarction: Myocardial Infarction-Net (MyI-NET)

As reported by the Association of American Medical Colleges (AAMC), in the US there will be an urgent shortage of physicians (approximately 122,000 by 2032) while the nation’s population is still growing and aging [21]. A similar situation is expected to emerge in the UK as only 2% of radiology departments have the ability to fulfill their imaging interpretation tasks within work hours, as reported by the Royal College of Radiologists (RCR) in “Clinical Radiology U.K. Workforce Census Report 2018” [22]. Meanwhile, the report also highlighted that only 2% of trusts and health boards in the UK have adequate interventional radiologists to provide for urgent procedures. Therefore, an automatic image interpretation service is urgently needed.

Deep learning has demonstrated great potential in biomedical data analysis with its powerful and advanced learning abilities [23,24,25]. For example, Nam et al. [26] reported in 2018 that their proposed algorithm for malignant pulmonary nodules based on deep learning outperformed the radiologist in radiograph classification. Our pilot work based on CNN [27] also illustrated that deep learning can be used for the automatic detection of MI. Here we make another step forward to improve the performance of MI detection based on machine learning by proposing a new class of models: MyI-Net. 

In order to optimize performance, we propose a new class of appropriately engineered deep leaning models. These models combine initial feature extraction (realized through ResNet and MobileNet-based models) followed by atrous spatial pyramid pooling (ASPP) to adjust the receptive field to preserve more image context. New feature maps are generated via fusing high-level features from ASPP and low-level features from one specific layer of the corresponding networks. Finally, the segmentation result is obtained via up-sampling to eventually recover the spatial information using an add-on module.

In order to deal with the other source of uncertainty, the issue of inherently unbalanced datasets (the number of pixels corresponding to scarred tissue in an image is always considerably smaller than that of the pixels corresponding to muscle, background, or blood pool) while fully using all the data, we use an appropriately constructed weight matrix. As training datasets are always limited, and in order to increase robustness, we propose three different augmentation methods integrated in this model to make a diversified dataset for training. New models as well as their state-of-the-art counterparts, which were used as baseline comparisons, were trained and validated on 1822 unique MRI images collected in our lab from research patients with MI. Details of the proposed new class are provided below.

**Figure 1:**
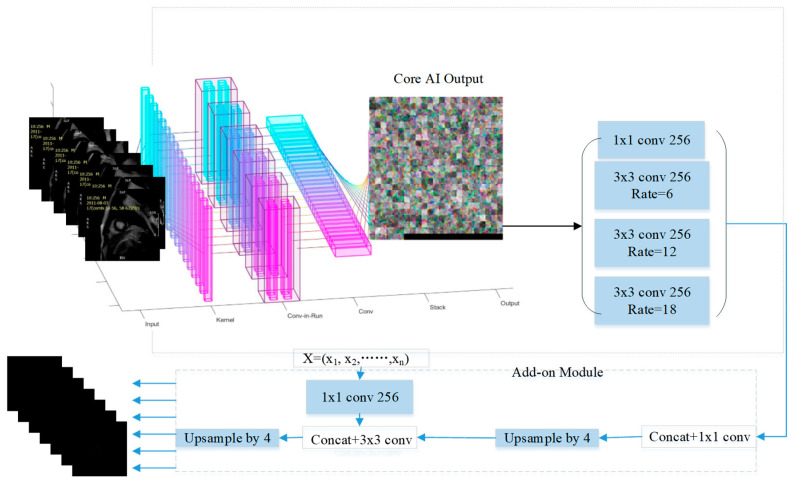
Flowchart of the proposed model.

At the core of this new model class is the proposal to exploit a wealth of deep learning architectures whose efficiency has already been demonstrated in image processing applications. We use these models as a part of the feature extraction process. Feature extraction is then combined with atrous spatial pooling—ASPP. The latter generates multiple receptive fields, enabling us to catch information at different spatial scales in a balanced way. This is followed by an add-on module for spatial information recovery. The process, applied to MRI MI segmentation, is illustrated with a diagram shown in Figure 1. In Figure 1, $X=\left(x_{1},x_{2},\ldots ,{\;x}_{n}\right)$ stands for the low-level features that can be extracted from the specific i-th layer of the base feature extraction network. Core outputs of the backbone deep learning model that are used in the initial processing pipeline are referred to as the high-level features.

### 3.1. Feature Extraction via MI-ResNet

**Figure 2:**
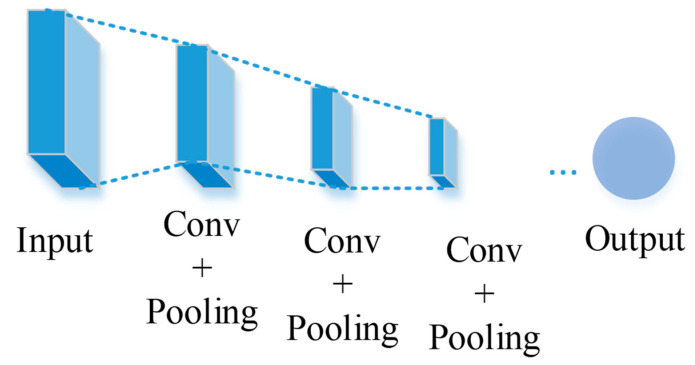
Structure of conventional CNN.

**Figure 3:**
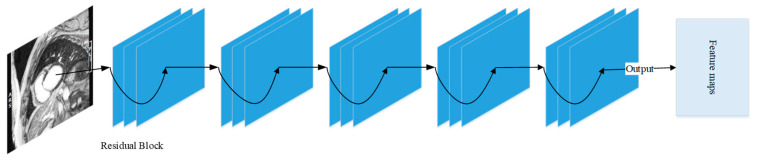
Structure of MI-ResNet.

**Figure 4:**
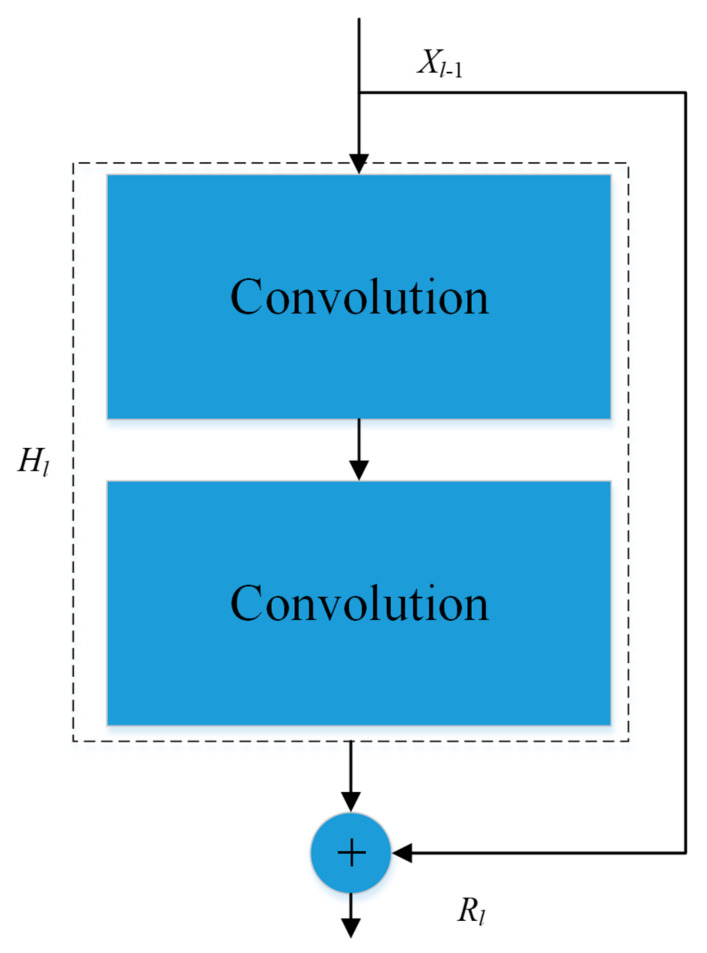
Short-cut structure of the ResNet block.

Feature extraction is based on deep CNN networks (see e.g., [26,28,29]). Figure 2 shows an example flowchart of relevant processes in a conventional CNN. As is shown in Figure 2, all layers, including convolutional layers, ReLu layers, pooling layers, are cascaded gradually. However, such simple and uniformly cascaded structures have severe technical drawbacks. Particularly, it may be hard to train deep conventional CNNs in practice due to the well-known problems of either gradient exploding or gradient vanishing. To circumvent this issue, here we adopt the ResNet model of CNN proposed by He et al. [30] as the basic backbone model for feature extraction. We call this backbone model MI-ResNet as shown in Figure 3. In contrast to conventional CNNs, ResNet provides a structure with short-cut connections by skipping one or more weight layers, as shown in Figure 4.

Mathematically, the structure of CNN and ResNet processing blocks can be expressed as:

(1) $$\mathrm{CNN}:\;R_{l}=H_{l}\;$$

(2) $$\mathrm{ResNet}:\;R_{l}=H_{l}+x_{l-1}$$

in which $x_{l-1}$ stands for the output from the previous layer, *H_l_* is the output of the *l*-th layer in the conventional CNN’s counterpart, and $R_{l}$ is the output of a ResNet constructed from the original CNN by adding a short-cut connection (residual information).

### 3.2. Feature Extraction via MI-MobileNet

**Figure 5:**
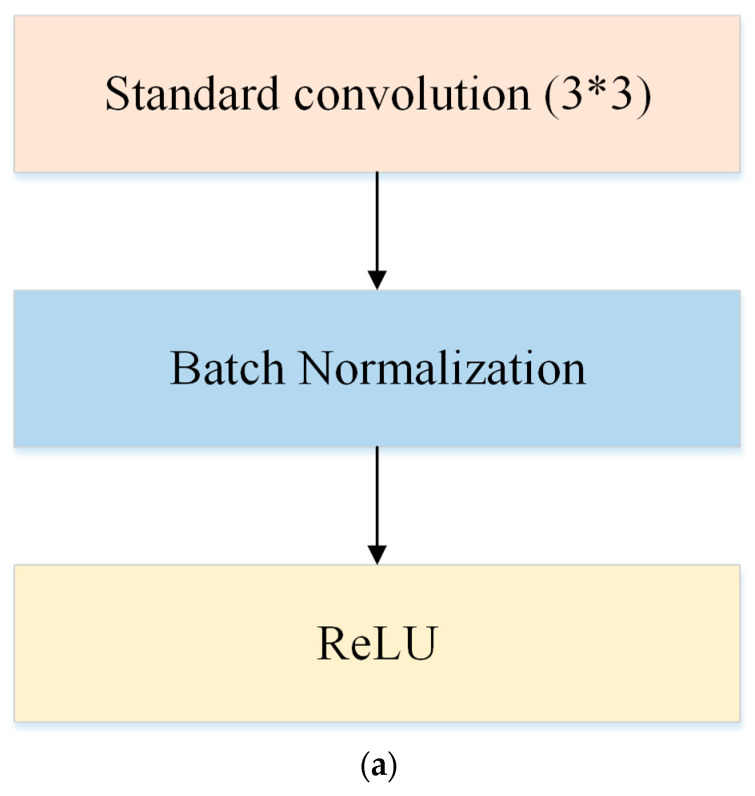

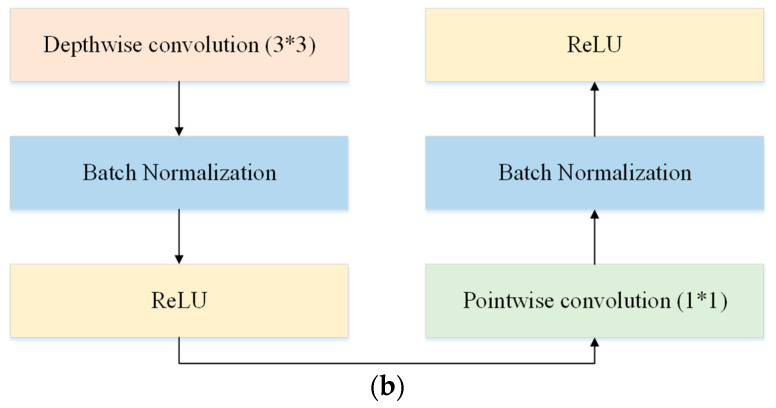
The structures of standard convolution and DSC. (**a**) Standard convolution. (**b**) DSC.

ResNet architectures mainly focus on improving the accuracy of the deep network and ignore computation costs. Therefore, we consider MobileNetV2 as another potentially relevant backbone for our proposed MyI-Net. We call such architectures MI-MobileNet feature extractors. MobileNetV2 was proposed by Sandler et al. [31], a research group at Google. Before the advent of MobileV2, MobileNet was first introduced by Howard et al. [32] also at Google with the idea of depthwise separable convolution (DSC), which can dramatically reduce the model size and complexity. DSC can be described by two components: depthwise convolution (DC) and pointwise convolution. DC applies a single filter to each input channel and pointwise convolution applies 1 × 1 filters to create a linear combination of the output of DC layers. There also are batch normalization layers and ReLu layers to follow both the DC layer and pointwise convolution layer. The structure of DSC is shown in Figure 5. As the DC in MobileNet used the 3 × 3 filter, we therefore used the 3 × 3 filter in Figure 5 to show the difference between the structures of the standard convolution and DSC. 

Though MobileNet is rather small and computationally cost-efficient, to make it more flexible in practical applications with the requirement of faster running and smaller structure, MobileNet utilizes the idea of the so-called width and resolution multipliers. The width multiplier makes the network uniformly thin at each layer, and the resolution multiplier is applied to the input image to further reduce the computation cost. The detail is introduced as follows: 

Suppose that the width multiplier is ε, then for a given layer with the number of input channels $D_{I}$, the number of input channels becomes ${\varepsilon D}_{I}$. Likewise, if $D_{O}$ is the number of output channels, then this layer’s number of output channels will become $\varepsilon D_{O}$. Therefore, the computation cost of one DSC can be reduced to:

(3) $${\mathrm{Cos}t}_{\mathrm{DSC}}={\varepsilon D}_{I}{*D}_{F}{*D}_{F}+{\varepsilon D}_{I}{*\varepsilon D}_{O}{*D}_{F}{*D}_{F}$$

where $D_{F}$ stands for the spatial width and height of a square input feature map, and ε is taken in the interval $\left(0\;1\right]$. If we set the resolution multiplier equal to $\delta \in \left(0\;1\right]$ as well, then the computation cost can be described as:

(4) $${\mathrm{Cos}t}_{\mathrm{DSC}}={\varepsilon D}_{I}*\delta D_{F}*\delta D_{F}+{\varepsilon D}_{I}{*\varepsilon D}_{O}*\delta D_{F}*\delta D_{F}$$

In summary, MobileNet replaces the standard convolution using DSC with batch normalization and ReLU excluding the first full convolution layer. In addition, it enables further reduction of the overall computation costs by using appropriate width resolution multipliers.

**Figure 6:**
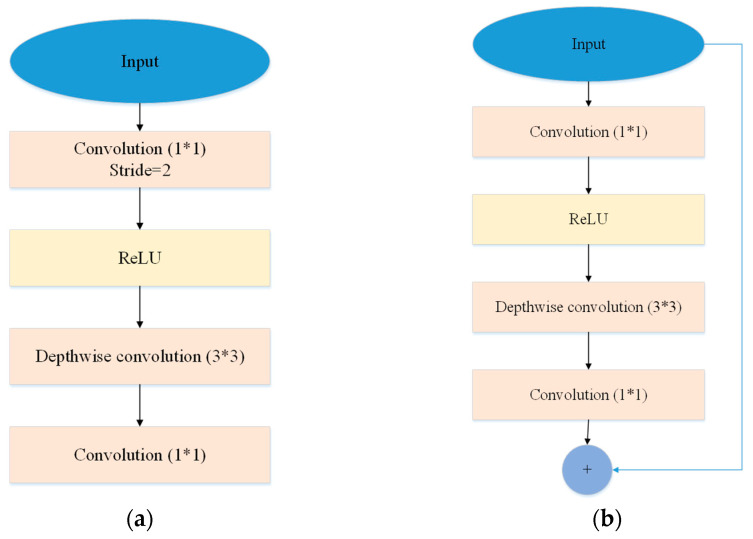
Residual block and down-sizing block in MobileNetV2. (**a**) Residual block (Stride = 1) (**b**) Down-sizing block (Stride = 2).

MobileNetV2 architecture is a descendant of the base MobileNet in which further processing operations are added, namely bottlenecks. For the bottlenecks, there are two types of blocks: residual block and down-sizing block, as shown in Figure 6a,b, respectively. As shown in Figure 6, a bottleneck in MobileNetV2 is characterized by the first layer being the 1 × 1 convolution layer followed by ReLU6, the second layer being the DC layer and the final layer being the 1 × 1 convolution layer without any non-linear operation. In the residual block, the input of the corresponding block is combined with the output of the final 1 × 1 convolution layer. The whole structure of MobileNetV2 can be found in [31]. For feature extraction, MI-MobileNet takes the raw LGE-CMR image and generates corresponding features following the MobileNetV2 processing flowchart.

### 3.3. Atrous Spatial Pyramid Pooling

**Figure 7:**
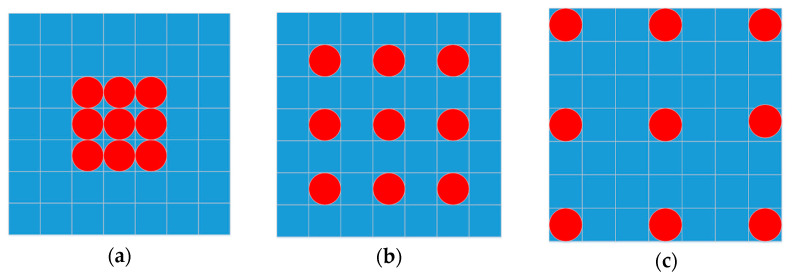
Atrous convolution (the red dot means non-zero). (**a**) Conventional convolution. (**b**) Dilation convolution(k = 2). (**c**) Dilation convolution(k = 3).

In the conventional convolution neural network, we can obtain more low-level and high-level features while the network goes deeper and wider. However, the problem is that this standard approach produces a relatively limited number of spatially local convolutional features. The latter, however, may contain crucial information for semantic segmentation. For semantic segmentation, conventional approaches therefore employ different methods to increase spatially relevant information content, such as stacking more layers and up-sampling. Theoretically, the amount of spatially relevant information can be increased through a broader spectrum of convolutional filters used in the network, from small to large. The size of these filters is sometimes referred to as a receptive field. Thus, the overall receptive field sizes can be increased if we stack more layers. However, not all information in the receptive fields is equally effective or useful. Likewise, up-sampling increases the receptive field but at the same time may negatively affect our capability to extract useful information about the local context. In order to keep the context information, which is essential, and decrease the ambiguity caused by local areas while keeping the number of parameters in the receptive fields constant, atrous convolution, also called dilated convolution, was proposed [33]. Atrous convolution was implemented via assigning zero values to the relevant weights of the filter. Formally, it can be expressed as:

(5) $$h\left(i\right)=\overset{N}{\underset{n=1}{\sum }}f\left(i+kn\right).w\left(n\right)$$

where k stands for the dilation rate. When k=1, it reverts to a conventional convolution. $w\left(n\right)$ represents the filter with size n, $f\left(i\right)$ is the input and $h\left(i\right)$ is the output of the atrous convolution. Figure 7 shows examples of the atrous convolution with rate *k* = 1, 2, 3. When *k* = 2, 3, we obtain feature maps with larger receptive field, as shown in Figure 7b,c.

**Figure 8:**
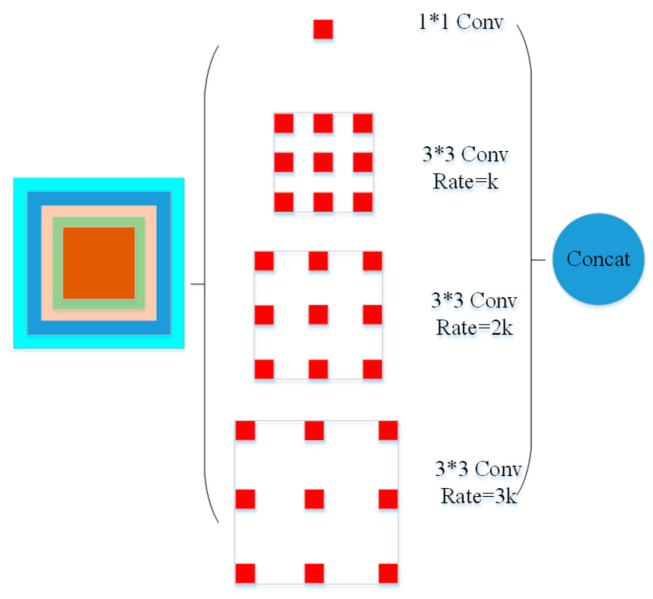
Structure of atrous spatial pooling with feature extraction network.

In this paper, we propose using the atrous spatial pyramid pooling (ASPP) method as an extra module cascaded to the feature extraction network as shown in Figure 1. This extra module enables us to adjust and maintain constant size (weight-wise) of receptive fields across scales in the network. ASPP feature maps are generated via a 1 × 1 convolutions and three atrous convolutions with rate *k*, 2*k* and 3*k*. In the models we generated in this work, the value of *k* was set to 6. Their outputs were then fused together to form new feature maps as shown in Figure 8.

### 3.4. Weight Matrix

**Figure 9:**
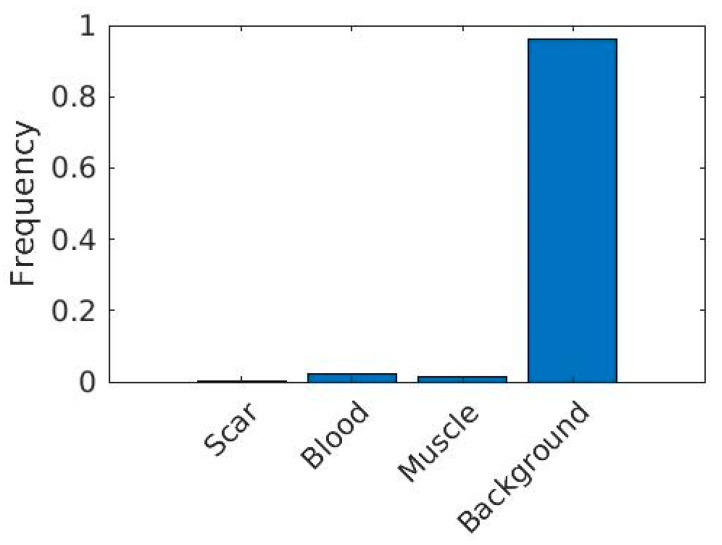
Statistical data of each element in the raw images.

Figure 9 shows the class frequencies of the data in our dataset. As we can see from this figure, the dataset is severely imbalanced. Imbalanced datasets, if processed without due care, could produce models that are biased towards the most common category.

In order to avoid this problem and at the same time to utilize our data fully, we employ an appropriately chosen weight matrix to balance the contribution of data from different-sized classes whilst training the model. 

The weight matrix assigns appropriate weights to each training sample when a training algorithm computes and subsequently uses a given loss function. In this work, the highest weight was assigned to data associated with the LGE tissue, and smallest weight was assigned to data samples representing background pixels. Mathematically, the weight matrix we used is defined as follows:

(6) $$F_{i}=\frac{N_{i}}{\sum_{i=1}^{n}N_{i}}\;,i=1,2,\ldots ,n$$

(7) $$W_{i}=\frac{Median\left\{F_{i}\right\}}{F_{i}}$$

where $N_{i}$ represents the number of pixels in each class of the dataset, *n* represents the total number of categories/classes, $F_{i}$ stands for the frequency, i represents indices, and $W_{i}$ is the weight of each category/class.

### 3.5. Data Augmentation

**Figure 10:**
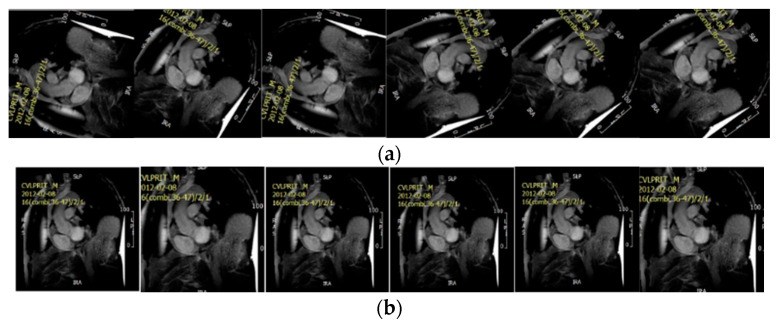
Examples of data augmentation. (**a**) Random rotation within [0, 360]. (**b**) Random scaling within [0.9, 1.1].

As the dataset to train, test, and validate our models was very limited (particularly for LGE pixels), data augmentation was utilized to produce a more diversified dataset. The data augmentation methods used in this work include geometric transformations, such as rotation (from 0 to 360 degrees at random) and random scaling with scaling factors from 0.9 to 1.1 for the training dataset in Figure 10.

### 3.6. Performance Metrics

In order to validate the proposed methods, we employed different performance metrics, including accuracy, bfscore, IoU, and per-image score.

Accuracy: Accuracy at the pixel level was defined as the percentage of correctly identified pixels for each category, which is used by most semantic segmentation. Suppose the confusion matrix *P*, which stands for all the prediction results for the whole dataset is:

(8) $$P_{ab}=\sum_{I\in D}\left|\left\{z\in I\;and\;S_{g}^{I}\left(z\right)=a\;and\;S_{p}^{I}\left(z\right)=b\right\}\right|$$

where z stands for each pixel in the image *I* and $S_{g}^{I}\left(z\right)$ stands for the ground truth and $S_{p}^{I}\left(z\right)$ is the prediction result for *z*. $P_{ab}$ is the total number of pixels with label a and prediction output b. If we have n categories, then we can obtain $M_{a}$=$\;\sum_{b=1}^{n}P_{ab}$ as the total number of pixels with label a. $H_{b}=\sum_{a}P_{ab}$ as the number of pixels predicted as *b*. Then the global accuracy can be expressed as:

(9) $$gAcc=\frac{\sum_{a=1}^{n}P_{aa}}{\sum_{a=1}^{n}M_{a}}$$

Category accuracy was the total fraction of correctly detected pixels in that category. The global accuracy gAcc  was the fraction of all correctly detected pixels regardless of the category information, which can provide a quick and inexpensive measure of the segmentation algorithm. The mean accuracy was the average category accuracies:

(10) $$aAcc=\frac{1}{n}\sum_{a=1}^{n}\frac{P_{aa}}{M_{a}}$$

Bfscore provided the information of how well the predicted boundary aligned with the ground truth boundary. As the contour quality contributed significantly to the segmentation result, therefore, in this research, we proposed using the bfscore as one measure, which is mathematically expressed as the harmonic mean of the recall $R_{o}$ and precision $P_{o}\;$ to determine whether the predicted boundary matches to the ground truth boundary with a distance error tolerance ∂. The detailed description is as follows: 

Let $B_{g}^{o}$ be the boundary of the binary ground truth segmentation map for a specific class *o* with $S_{g}^{o}\left(z\right)=⟦S_{g}\left(z\right)==o⟧$ and ⟦z⟧ be the Iverson bracket notation (2):

(11) $$⟦z⟧=\left\{\begin{array}{c} 1\;if\;z=true\\ 0\;otherwise \end{array}\right.$$

Let $B_{p}^{o}$ be the predicted binary contour map for the segmentation result $S_{p}^{o}$. Then, with a distance error tolerance ∂, precision and recall for each class are defined as

(12) $$P_{o}=\frac{1}{\left|B_{P}\right|}\sum_{z\in B_{P}^{o}}⟦d\left(z,B_{g}^{o}\right)<\partial ⟧$$

(13) $$R_{o}=\frac{1}{\left|B_{g}\right|}\sum_{z\in B_{g}^{o}}⟦d\left(z,B_{p}^{o}\right)<\partial ⟧$$

in which $d\left(\;\right)$ stands for the Euclidean distance and ∂ is usually set as 0.75% of the image diagonal.

Then, for the category *o*, we can obtain:

(14) $$F_{1}^{c}=\frac{2*P_{o}*R_{o}}{P_{o}+R_{o}}$$

To finally generate the bfscore for each image, we can average $F_{1}^{c}$ over all classes. Similarily, we can average bfscore of each image over the whole dataset to obtain the dataset’s bfscore.

Intersection over union (IoU), which is also known as the Jaccard similarity coefficient, can be utilized if we want to provide a statistical accuracy measure that helps to better reveal false positives. IoU was calculated by the ratio of correctly classified pixels to the number of ground truth and predicted pixels in that category. Weighted IoU (wIoU) was mainly used to measure the performance of the model tested on disproportionally sized classes, aiming to exclude the impact of errors in the small classes on the aggregate quality score.

(15) $$\mathrm{IoU}=\frac{1}{L}\overset{L}{\underset{a=1}{\sum }}\frac{P_{aa}}{M_{a}+H_{b}-P_{aa}}$$

However, as our dataset was severely imbalanced across categories, the mean IoU may not be an appropriate measure. Therefore, we used the wIoU instead of the mean IoU to measure the performance of the proposed algorithm.

(16) $$wIoU=\frac{1}{\sum_{a=1}^{n}\sum_{b=1}^{n}P_{ab}}\sum_{a=1}^{n}\frac{\sum_{b=1}^{n}P_{ab}P_{aa}}{\sum_{b=1}^{n}P_{ab}+\sum_{b=1}^{n}P_{ba}-P_{aa}}$$

Per image score: As we need to avoid developing an algorithm that works extremely well on some images but poorly on most images, it was necessary to check the performance of the model not only for individual pixels in our test set but also assess how the model worked for each individual image. Second, the per image score can help to reduce the bias towards the large objects, which is because the missing segmented least objects have a small impact on the confusion matrix. Third, per image score enables drawing realistic comparisons of image segmentation results produced by different algorithms with segmentation produced by clinical experts. Therefore, in this study, we also used the per image score as a performance metric.

## 4. Experiments and Results

### 4.1. Data Preparation

**Figure 11:**
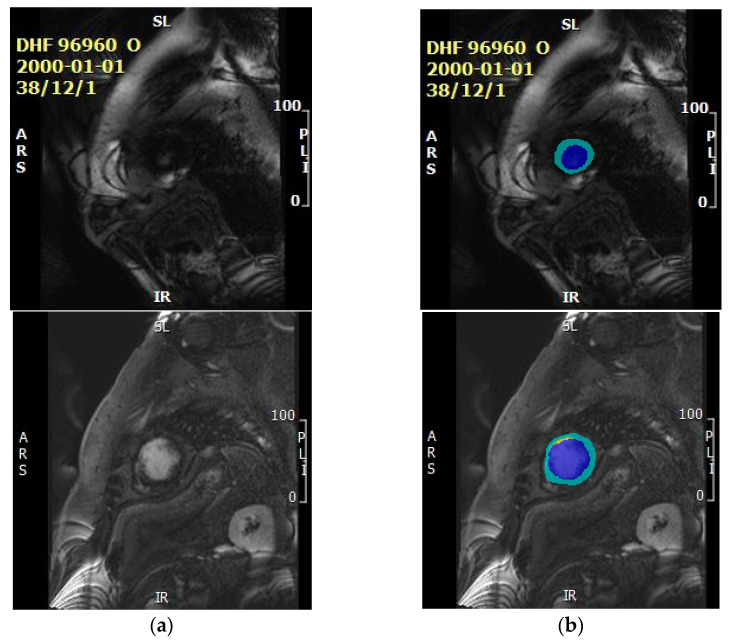
Examples of the raw image and labeled image. (**a**) Raw image. (**b**) Labeled image.

The data were collected from patients with MI. To date, we collected 1822 raw images that were manually annotated by human experts. The muscle, blood area, LGE, microvascular obstruction (MVO) and background were contoured by expert clinicians. The reference ground truth was then obtained as the employed experts manually labeled the raw images at the pixel level according to the contour information into the following five categories: background, blood, muscle, LGE and MVO using the Image Labeler integrated into MATLAB. In this research, the MVO and LGE areas were then combined, and both considered to be LGE due to the limited MVO data. The MRI images were resized to 256 by 256. Figure 11 shows two examples of the raw images (Figure 11a) and raw images with labels (Figure 11b). Unmasked areas in the images on the right represent the background, the blue areas highlight the blood pool, yellow areas indicate presence of LGE and green areas show the myocardium. Unfortunately, the sample raw image does not include the MVO area, as the MVO only appeared in very few images. After the data preparation, we divided the whole dataset into 60% for training, 20% for validation and the remaining 20% for testing. To avoid leakage of information from the training data into the test set, images in the tests set were taken from a cohort of patients whose MRI scans were not present in the training/validation sets.

### 4.2. Experiment Environment

All the experiments were carried out on a workstation with a 1.99 GHz processor and 16 GB memory with the Windows operating system. The proposed algorithm was implemented in Matlab without optimization. The training parameters are shown in Table 2. To make the comparison fair, we kept the training parameters the same as shown in Table 2. We trained the proposed model via the stochastic gradient descent method (SGDM). The initial learning rate was set as $e^{-3}$, the learning rate drop period was set to 10, and the learning rate drop factor was 3. The max number of epochs was 50, and the mini batch size was set to 10. All the algorithms were executed under the environment of GPU to accelerate the computing speed. The training algorithm would stop either because the max epochs were reached or the stop criteria were met, as we set the validation patience as 4.

### 4.3. Segmentation Result Based of Proposed Method

In order to explore the flexibility of our method and optimise performance, we built different models based on different feature extraction methods and named the three corresponding models MI-ResNet50-AC, MI-ResNet18-AC and MI-MobileNet-AC for easy remembering. Due to the data imbalance, the class weights were set as 13.7678, 0.7802, 1.3923 and 0.0163 for the LGE, muscle, blood and background, respectively, as is shown in Table 3. 

**Figure 12:**
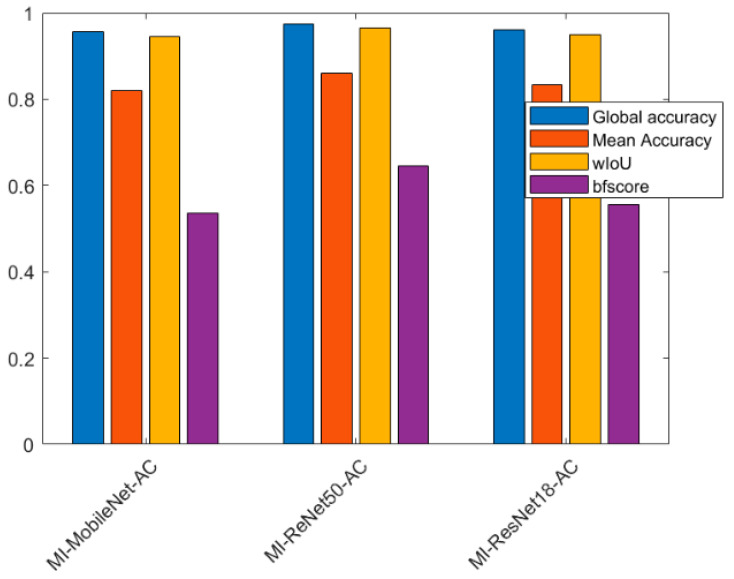
Bar chart of the performance of three proposed models.

As is seen in Table 4, for the global segmentation, MI-ReNet50-AC provides the best performance with global accuracy of 0.9738, mean accuracy of 0.8601, wIoU of 0.9647 and bfscore of 0.6446. MI-ResNet18-AC is slightly better than MI-MobileNet-AC. However, it is seen in Table 5, that for the scar tissue, MI-ResNet18-AC provides the best performance in terms of accuracy and similar performance in terms of bfscore compared with MI-ResNet50-AC. Figure 12 shows a bar chart for a clear performance comparison for all three proposed models.

Table 6 shows the confusion matrix based on each model for a clear performance comparison. The rows stand for the predicted class and the columns stand for the true class. The correctly classified categories are shown as the diagonal cells, and the incorrectly classified observations are shown as the off-diagonal cells.

The time analysis was based on the current training dataset. For the proposed three models, MI-MobileNet-AC, MI-ReNet50-AC and MI-ResNet18-AC, the time costs were 24′1″, 57′35″ and 24′50″, respectively.

Table 7 shows that the computational costs of MI-RestNet50-AC are double those of the other two methods. MI-ResNet18-AC and MI-MobileNet-AC have similar computation cost. With the validation patience as 4, MI-MobileNet-AC, MI- MI-ResNet50-AC, MI- MI-ResNet18-AC stopped at epoch 7, 2 and 10, respectively (all stopped earlier than the maximal number of epochs we set for these experiments). 

### 4.4. Segmentation Result Based on State of Art Methods

In order to demonstrate the advantage of the proposed approach and models, we compared the performances of our models to those of the state-of-the-art models, including conventional CNN and UNet (3). A summary of performance for these models in the task of global segmentation is shown in Table 8. 

As we can see from Table 8, our proposed model MI-ResNet50-AC provides the highest accuracy and bfscores (the Unet architecture trained on the same data provides global accuracy of 0.6332, mean accuracy 0.6222, with 0.6117 for the wIoU, and a bfscore of 0.1626). Remarkably, our network’s bfscore is approximately four-fold higher than that of the state of the art on our data. 

**Figure 13:**
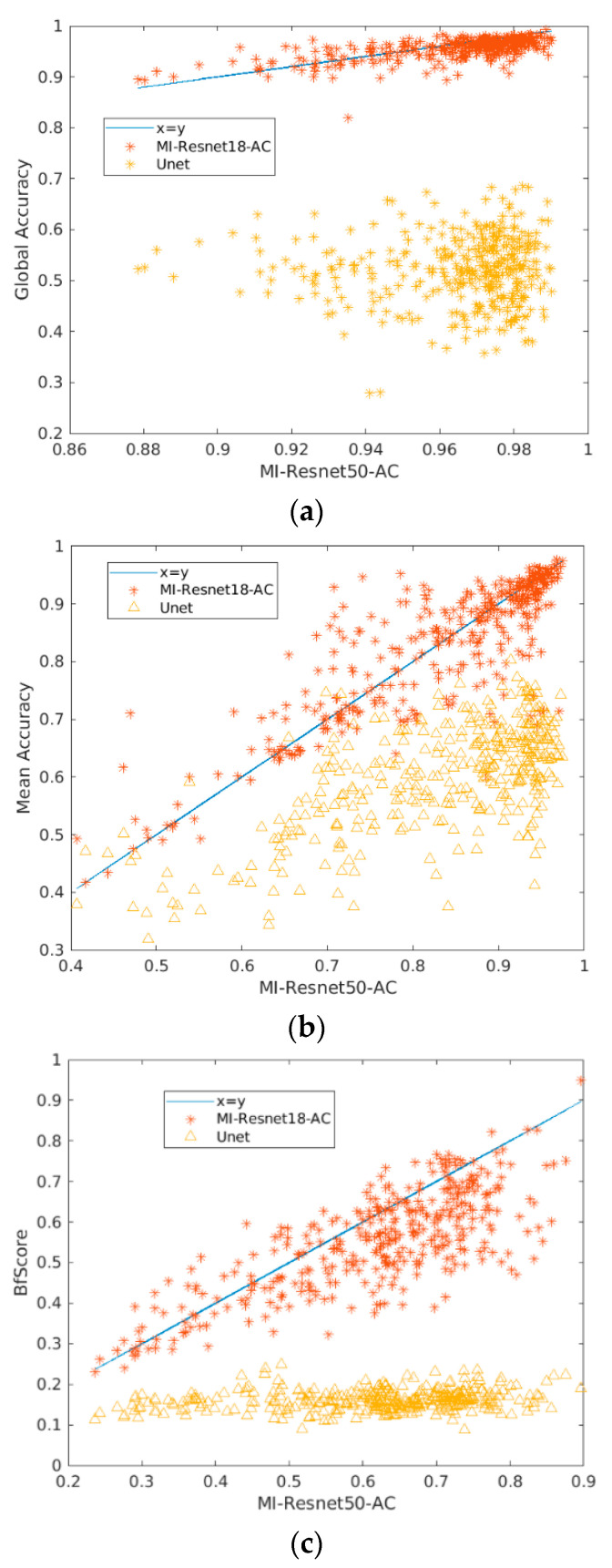
Bar scatter plot of the performance based on per image of MI-ResNet18-AC, UNet vs MI-ResNet50-AC. (**a**) Global accuracy. (**b**) Mean accuracy. (**c**) Mean accuracy.

In order to provide a clearer relation between the performance of our proposed method and the most recent method, Unet, we provide the scatter plot (based on per image comparison) shown in Figure 13. Figure 13a–c show the per-image global accuracy, average accuracy and bfscore comparison, respectively by MI-ResNet18-AC and Unet vs. the MI-ResNet50-AC. We can see that from the perspective of global segmentation for each image, MI-ResNet50-AC surpasses MI-ResNet18-AC in some cases. For a few cases, MI-ResNet18-AC performs better than MI-ResNet50-AC. However, both MI-ResNet50-AC and MI-ResNet18-AC outperform UNet for most images in this dataset in terms of global accuracy, average accuracy and bfscore.

**Figure 14:**
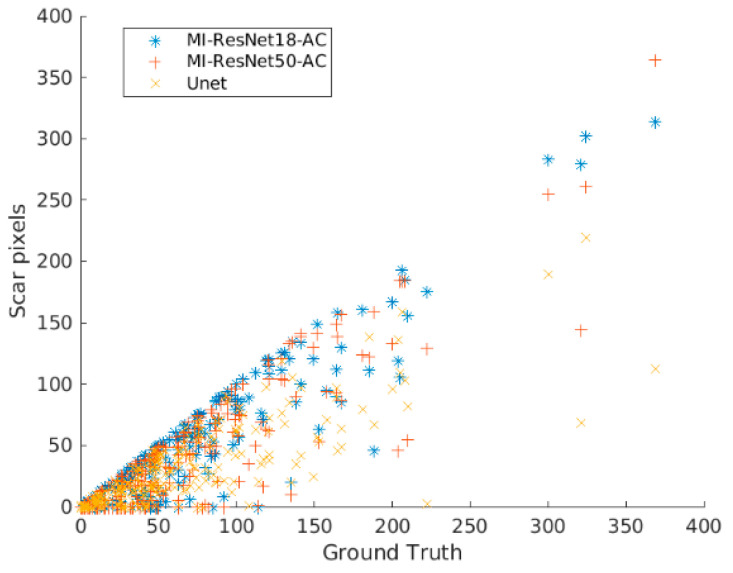
LGE detection based on MI-ResNet18-AC, MI-ResNet50-AC and UNet vs. the ground truth.

**Figure 15:**
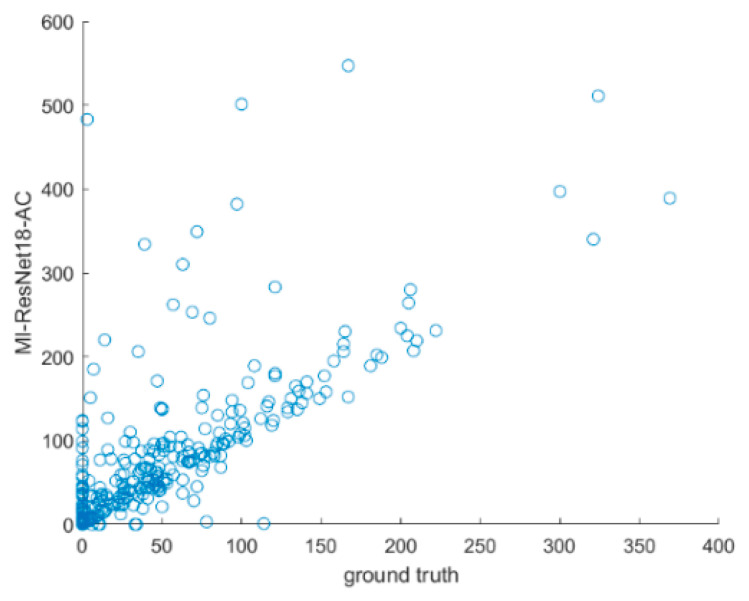
LGE detection based on MI-ResNet18-AC vs. the ground truth including false alarms.

**Figure 16:**
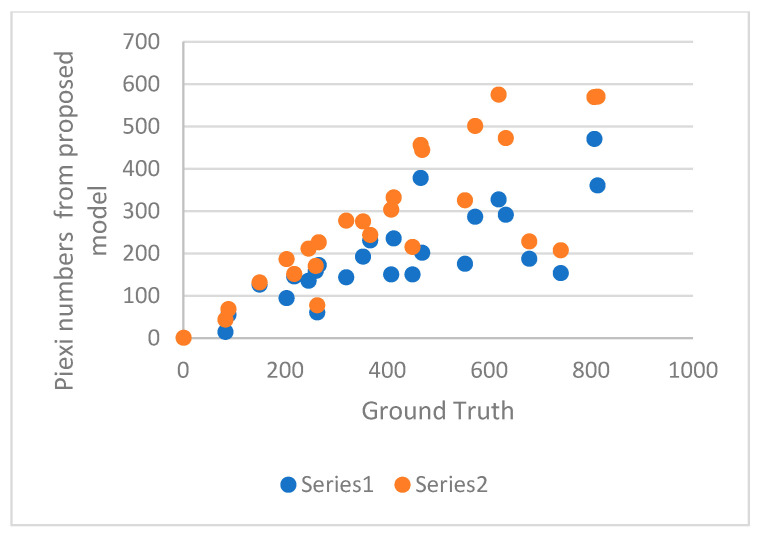
Detection result per case. Series 1 includes false alarms and series 2 only contains the true positives.

As can be seen from the confusion matrix (shown in Table 1) and Table 5, MI-ResNet18-AC has the best performance for LGE quantification compared to MI-ResNet50-AC and UNet. Figure 14 shows the scatter plot of the correctly detected LGE elements using MI-ResNet18-AC, MI-ResNet50-AC and UNet vs. the ground truth. Based on this figure, we can see that MI-ResNet18-AC obviously has more cases close to the diagonal line. Figure 15 shows the LGE detection including false alarms based on MI-ResNet18-AC vs. the ground truth, where we can see that though MI-ResNet18-AC can detect the true positives at a satisfactory rate, the false alarm rate is still relatively high for clinical applications. Therefore, in future research, we need to pay extra attention to reduce the false alarm rate. For clinical reference, we also show the scatter plots per case. It is apparent that further work may be needed to reduce false alarm rate as shown in Figure 16. Error correction approaches may potentially be used to address this issue [34,35,36]. Detailed exploration of such functionality, however, is outside of the scope of the current work.

## 5. Conclusions

### 5.1. A Short Summary of Results

In this paper, we proposed a new end-to-end method for automatic MI segmentation, as the detection and quantification of the MI are crucial for determining clinical management and prognosis. Although LGE-CMR [37] is the non-invasive “gold standard” method as it permits optimal differentiation between normal and damaged myocardium with the use of gadolinium-based contrast agents and special magnetic resonance pulse sequences, to date, there is no fully automatic “Gold standard” method for the detection and quantification of MI. In this work, we make a step forward towards achieving this aim with the hope of reducing the uncertainty brought by the technical variability and inherent bias of the data and labels. We propose a novel deep learning model, MyI-Net, which accommodates MI-ResNet and MI-MobileNet models as initial feature extractors and is equipped with ASPP with an add-on module for the recovery of the spatial information to compute the final segmentation output. Considering the limited size of the dataset, a data augmentation pre-processing step was integrated in our model construction pipeline. It is apparent from Figure 9 that our dataset was severely imbalanced, as it contained primarily background elements, followed, in descending order, by blood pool, muscle and LGE. A weight matrix was used to minimize our classifier’s bias towards any specific category. The performances of the algorithm are shown in Table 4, with the best performance for global segmentation being provided by MI-ResNet50-AC with a global accuracy of 0.9738, mean accuracy of 0.8601, wIoU 0.9647, and bfscore 0.6446. Compared with state-of-the-art methods (Table 8), our model outperformed state-of-the-art architectures on the dataset we could access. However, considering the detection of LGE, we found that MI-ResNet18-AC, being a smaller model than MI-ResNet50-AC, provides the highest LGE detection accuracy and a bfscore similar to that of MI-ResNet50-AC. Furthermore, we compared the computation cost for the three proposed models. Table 7 shows that MI-ResNet50-AC required the largest amount of time compared to the other two models, mainly because MI-ResNet50-AC is a deeper network. Considering the above summary, we integrated both MI-ResNet50-AC and MI-ResNet18-AC into our proposed system MyI-Net. In general, the choice of specific feature extractor model depends on a given task (e.g., LGE detection or global segmentation). 

### 5.2. Limitations and Directions for Future Work

One limitation of this work is the relatively small size of the dataset. To build more accurate, higher-performing models, larger datasets may be required. These datasets are also necessary for testing the system at the level of individual patients. Further data should be collected from a greater variety of scanner vendors to improve model generalization. Meanwhile, based on the per-image analysis, we can observe that the proposed model is not as stable as desired. Therefore, the algorithm needs to be further tuned to achieve more robust and stable performance. We are considering a fusion technique in the future to explore the potential of the proposed deep learning models. We will also consider error correction approaches [35,36,38] in our future research to further improve the performance of our proposed method to minimize false positives. 

The primary focus of this work is the development of an appropriate segmentation algorithm capable of utilizing relevant information across different spatial scales regardless of other clininically informative parameters such as detailed characterization of cases. In real clinical scenarios, this latter information can be crucial for reliable and stable diagnostics. Inclusion of this additional information and fine-tuning our tool to account for such important details is a natural step for future research in this area.

## Figures and Tables

**Table 1:** Demographic data.

Variables	Values	Number	Rate
	Stemi	52	17.28%
	Non-stemi	249	82.72%
Gender	Male	172	57.14%
	Female	129	42.86%
Age at MRI	90–99	3	1%
	80–89	11	3.65%
	70–79	44	14.62%
	60–69	77	25.58%
	50–59	74	24.58%
	49–49	56	18.60%
	30–39	35	11.63%
	20–29	1	0.33%
Average	57	Std	13.67

**Table 2:** Training parameters.

Name	Parameters
Training algorithm	Data
Learn rate drop period	10
Learn rate drop factors	3
Initial learn rate	$e^{-3}$
Max epochs	50
Mini batch size	10
Execute environment	GPU
Validation patience	4

**Table 3:** Class weight.

Type	Weight
Training algorithm	SGDM
Learn rate drop period	10
Learn rate drop factors	3
Initial learn rate	$e^{-3}$
Max epochs	50
Mini batch size	10
Execute environment	GPU
Validation patience	4

**Table 4:** Performance achieved by the proposed models.

Model	Global Accuracy	Mean Accuracy	wIoU	Bfscore
MI-MobileNet-AC	0.9569	0.8202	0.9463	0.5351
MI-ResNet50-AC	0.9738	0.8601	0.9647	0.6446
MI-ResNet18-AC	0.9679	0.8483	0.9584	0.5839

**Table 5:** Performance for each category based on proposed models.

	Category	LGE	Blood	Muscle	Background
MI-ResNet50-AC	Accuracy	0.6429	0.8402	0.8779	0.9686
	bfscore	0.4634	0.6837	0.4022	0.8552
MI-ResNet18-AC	Accuracy	0.7441	0.8255	0.8511	0.9724
	bfscore	0.4221	0.6226	0.4187	0.8559
MI-MobileNet-AC	Accuracy	0.4245	0.8809	0.8567	0.9664
	bfscore	0.3669	0.5996	0.3729	0.8411

**Table 6:** Confusion matrix based on proposed models.

		Target Class			
		LGE	Blood	Muscle	Background
MI-ResNet50-AC Output class	LGE	0.6429	0.1557	0.1982	0.0031
	Blood	0.0543	0.8402	0.0980	0.0074
	Muscle	0.0352	0.0640	0.8779	0.0229
	Background	0	0.0016	0.0291	0.9686
MI-ResNet18-AC Output class	LGE	0.7441	0.1180	0.1371	0
	Blood	0.0774	0.8255	0.0904	0.0068
	Muscle	0.0676	0.0621	0.8511	0.0193
	Background	0.0023	0.0023	0.0230	0.9724
MI-MobileNet-AC Output class	LGE	0.4245	0.3429	0.2326	0
	Blood	0.0242	0.8809	0.0870	0.0079
	Muscle	0.0266	0.0964	0.8567	0.0203
	Background	0.0011	0.0051	0.0273	0.9664

**Table 7:** Time analysis of the proposed algorithm.

Model	Training Time Cost
MI-MobileNet-AC	24′1″
MI-ReNet50-AC	57′35″
MI-ResNet18-AC	24′50″

**Table 8:** Comparison to state-of-art methods.

Model	Global Accuracy	Mean Accuracy	wIoU	Bfscore
CNN	0.6021	0.5632	0.4367	0.1574
MI-ResNet50-AC	0.9738	0.8601	0.9647	0.6446
Unet	0.6332	0.6222	0.6117	0.1626

## Data Availability

Not applicable.
